# CDC Grand Rounds: Public Health Practices to Include Persons with Disabilities

**Published:** 2013-08-30

**Authors:** Georges Benjamin, Monika Mitra, Catherine Graham, Gloria Krahn, Stephen Luce, Michael Fox, Jennifer Hootman, Neelam Ghiya, Tanja Popovic

**Affiliations:** American Public Health Association; Univ of Massachusetts; Univ of South Carolina School of Medicine; Div of Human Development and Disability, National Center on Birth Defects and Developmental Disabilities; Div of Population Health, National Center for Chronic Disease Prevention and Health Promotion; Office of the Associate Director for Science, CDC

“Persons with disabilities” is a vague designation that might not always be understood (1,2). Persons with disabilities are persons with limitations in hearing, vision, mobility, or cognition, or with emotional or behavioral disorders. What they have in common is that they all experience a significant limitation in function that can make it harder to engage in some activity of daily living without accommodations or supports (3–5).

According to the World Health Organization, disability has three dimensions: 1) impairment in body function or structure, such as loss of a limb or loss of vision; 2) limitation in activity, such as difficulty seeing, hearing, walking, or problem solving; and 3) restriction in participating in normal daily activities, such as preparing a meal or driving a car. Any of these impairments, limitations, or restrictions is a disability if it is a result of a health condition in interaction with one’s environment (6).

These limitations all relate to health conditions experienced within the environment in which persons live, as well as to other personal factors. Environmental barriers can be physical barriers, such as stairs; communication barriers, such as websites that can’t be read by screen readers; discriminatory policies, such as restrictions on participation in physical activity programs; or societal attitudes, such as presumptions that persons with disabilities cannot be productive employees. Consequently, disability is not a health condition itself, but is the limitation viewed in the context of the community and society in which the person lives. Societal and environmental accommodations are therefore critical if persons with disabilities are to participate in public health programs that prevent disease and promote health (7).

## Disabilities in the United States

Based on U.S. Department of Health and Human Services disability data standards released in 2011 that consider only serious limitation, about 16% of U.S. adults, or 37.5 million, have a disability (8). Disability-associated health-care expenditures have been estimated at nearly $400 billion in 2006, more than a quarter of all national health expenditures for that year (9). Although persons with disabilities have similar needs for eating healthful foods, being active, managing stress, and having regular medical checkups as persons without disabilities, as a population they have higher rates of poverty, social isolation, and other social determinants that can make it more difficult to access health and public health services (6,7).

**FIGURE 1:**
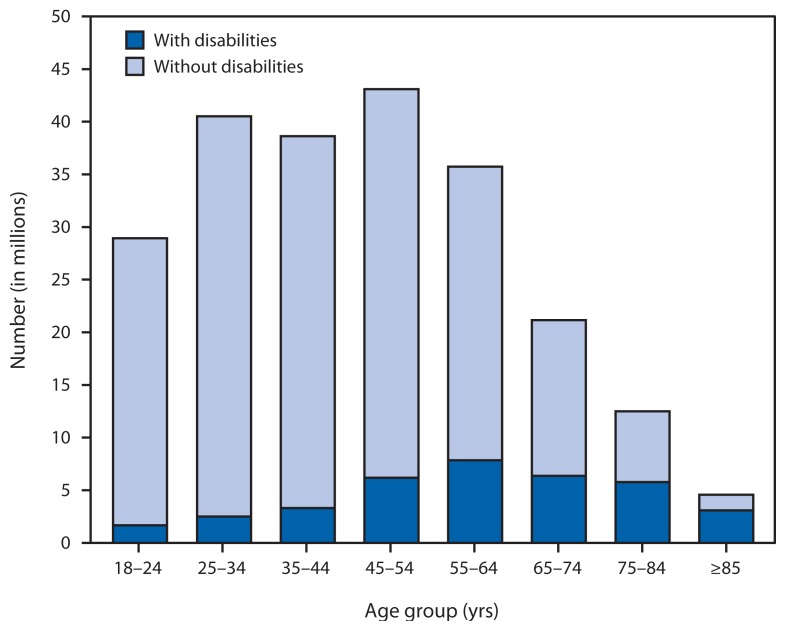
Number of adults with and without disabilities,* by age group — National Health Interview Survey, United States, 2010 * Weighted population estimates.

**FIGURE 2:**
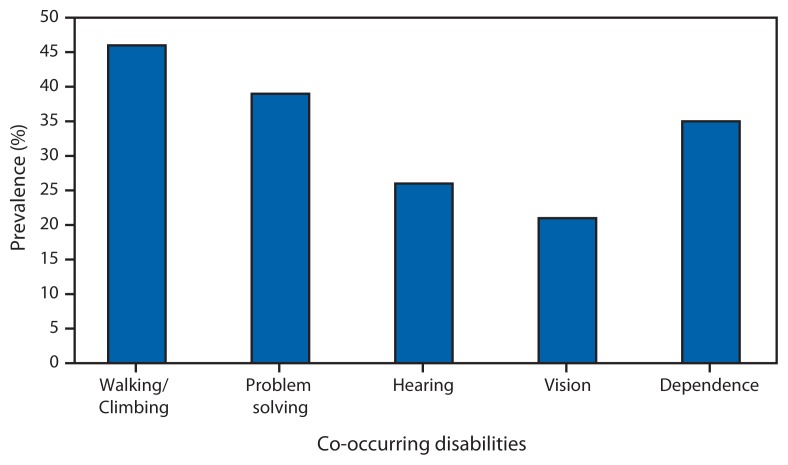
Prevalence of disability types among adults with co-occurring disabilities — National Health Interview Survey, United States, 2010–2011

**FIGURE 3:**
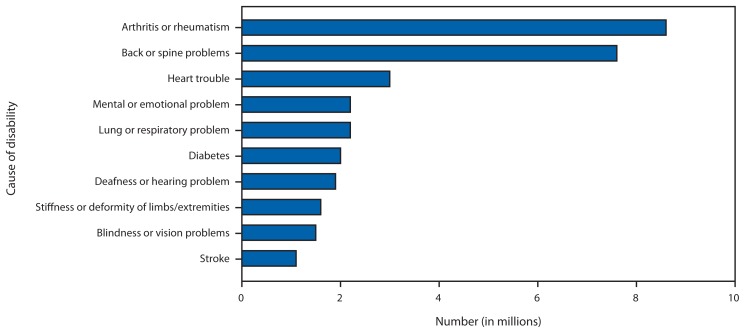
Top 10 causes of disability among adults — Survey of Income and Program Participation, United States, 2005

The estimated proportion of adults with disabilities increases with age, and more than one third of all adults with disabilities are aged 45–64 years (Figure 1). Persons experience different types of activity limitations, with the most often occurring limitations involving walking/climbing, problem-solving, hearing, seeing, and dependency on another individual (Figure 2). In addition, 43% of persons reporting disabilities report having more than one limitation. The health conditions that respondents identify most often as the main causes of their disability are arthritis and back problems, followed by heart problems, respiratory problems, emotional problems, diabetes, hearing problems, limb problems, vision problems, and stroke (Figure 3) (3).

## Disparities in Health Among Persons with Disabilities

The Americans with Disabilities Act of 1990 was the first civil rights law in the United States that specifically addressed the needs of persons with disabilities (10). Eliminating disparities between persons with and without disabilities was given a focus in *Healthy People 2010* as a preventable outcome of disease or illness (11). However despite these efforts, persons with disabilities continue to face significant health disparities. Approximately 39% of adults with disabilities in the United States reported experiencing fair to poor health based on a 5-level health status question, compared with fewer than 9% of adults without disabilities (12). Obesity rates for children with disabilities in the United States are approximately 38% higher than for children without disabilities (13).

**FIGURE 4:**
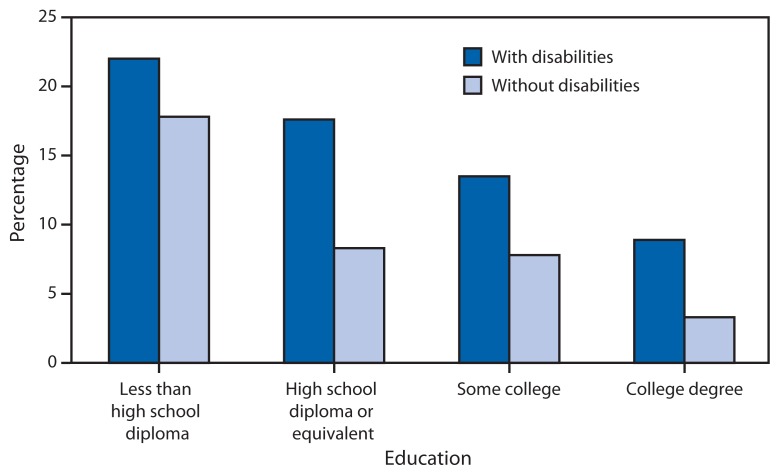
Percentage of adults with and without disabilities reporting cost as a barrier to seeking health care, by education — Behavior Risk Factor Surveillance System, Massachusetts, 2010

The example of Massachusetts illustrates the disparities gap at the state level. Persons with disabilities in Massachusetts and in the United States overall are more likely to report experiencing >14 days of poor mental health in the past month compared with those not reporting a disability. Almost 25% of adults with disabilities report poor mental health, compared with 6% of adults without disabilities. In Massachusetts, 22% of adults with disabilities report smoking, compared with 13% of adults without disabilities. Persons with disabilities are also much more likely to report not seeing a doctor because of expense, regardless of their education level (Figure 4). In addition, men and women with disabilities are at a heightened risk for lifetime and current sexual violence victimization, and women with disabilities are at a greater risk for lifetime sexual violence, lifetime completed and attempted rape, and sexual violence in the past year (14–16). Current research elsewhere suggests that persons with mental illness and intellectual disabilities are also at greater risk for violence victimization compared with those with other disabilities (17–20).

## Public Health Strategy: Making the Broadest Impact

Prevention of disabilities has been the focus of public health, and prevention remains its primary focus, but as disability is acknowledged as part of the normal human experience, a secondary focus of public health has become the promotion of the health of persons with disabilities by identifying and closing reducible gaps between the health of persons with and without disabilities (7).

CDC and other public health organizations can achieve the broadest impact by 1) including persons with disabilities in mainstream programs and services wherever possible; 2) using approaches that are common to all types of disabilities to address the unique health needs of persons with disabilities, such as physical barriers in their environment; and 3) using a condition-specific focus where that is essential because the problem is unique to persons with that condition (21).

CDC is including persons with disabilities in its surveys, programs, policies, and communications. It funds a network of 18 state disability and health programs that work within their states to improve health-care access, health promotion, and emergency preparedness, as well as five National Public Health Practice and Resource Center Programs to reach key populations with health communications and interventions (22). These centers address intellectual disabilities, limb loss, paralysis, select mental health disorders, and physical activity.

Five strategies are employed in this work: 1) promoting the inclusion of standardized disability identifiers in data collection instruments; 2) advancing research that increases understanding of health disparities associated with populations with disabilities; 3) identifying and helping to develop evidence-based interventions for persons with disabilities; 4) training health-care and public health professionals about the needs of persons with disabilities; and 5) helping to create barrier-free environments to ensure that health-care offices, medical and diagnostic equipment, health surveys, gyms, and the community at large are accessible. Inclusion and meaningful involvement of persons with disabilities in the development and implementation of all public health programs underlies each approach (21).

Arthritis, the most frequent cause of disability, is one of the most common chronic diseases, affecting quality of life for about 50 million U.S. adults and 300,000 children. Forty percent of adults with arthritis are limited in their usual activities, 33% report severe pain, and 11% are restricted in valued social activities. These factors contribute to poor quality of life (23). By 2030, there will be 67 million adults with arthritis, and 25 million of them will be limited in their usual activities. These estimates are conservative because they only take into consideration the aging of the population and do not consider the current prevalence of obesity, which is expected to add to the prevalence of arthritis still further. CDC funds 12 state health departments to deliver physical activity and self-management education programs to adults with arthritis in local communities. These programs have been proven to help persons decrease pain, increase function and quality of life, and maintain independence (24,25).

Public health organizations should serve as exemplars regarding inclusion of persons with disabilities in all aspects of their activities. A good example is the American Public Health Association (APHA), the nation’s oldest and largest organization of public health practitioners. APHA has a Disability Section, which has added disability issues to APHA’s broader policy agenda. In addition, APHA has put in place activities that support access for persons with disabilities at their annual scientific meeting. Measures include accessible facilities at the meeting venues, accessible web pages, and provision of accessibility resources and services, sign language interpreters, Americans with Disabilities Act training for hotel staff and vendors, an accessibility desk in each meeting venue, an accessibility guide to each convention city, on-call accessible shuttle van and regular shuttle buses with lifts, reimbursement for taxi service for registrants with mobility limitations, and assistive listening devices. Ultimately, the goal is to improve access to knowledge for APHA members and the broader public health community, decrease costs for preventable conditions among persons with disabilities, and improve quality of care and health outcomes for the entire public (26).

## Public Health Interventions at the State Level: South Carolina

Physical access to health-care services is a vexing problem for many persons with physical disabilities because they must overcome numerous obstacles even before they can receive care in a physician’s office, such as parking, entering the building, going to an examination room, and using the restroom. The CDC-funded South Carolina Disability and Health Program (SCDHP) assessed this problem with the goal of improving accessibility of primary-care sites. Under SCDHP leadership, the health department’s Best Chance Network breast and cervical screening program assisted in recruiting participant sites for assessments. The Office of Rural Health also recruited participant sites for assessments and offered low-interest loans to those sites for modifications. SCDHP assessed 150 sites with a patient load of over 750,000 and provided recommendations for changes, using additional funding from a state insurance provider to provide mini-grants to facilities to make accommodations. This led to almost one third of practices making changes related to parking areas, ramps, doors, restrooms, signage, equipment, and accessibility to the equipment.

SCDHP is involved in several aspects of work on obesity prevention (27). The state has utilized an evidence-based program called Steps to Your Health designed specifically for persons with disabilities. This 8-week participatory program covering healthy eating and physical activity has drawn over 5,200 participants using a train-the-trainer model. Results indicated that participants had a weight loss of ≥5 pounds (≥2.3 kg) and an increase in knowledge of healthy food choices (28). Beginning in 2012, SCDHP began collaborating with the Arthritis Foundation Exercise program to extend this approach further using state-sponsored senior centers and disability service providers.

Finally, emergency preparedness was considered especially critical because South Carolina is a coastal, hurricane-prone, rural state with a high level of poverty. An emergency planning committee for persons with functional needs was formed with diverse stakeholders, including SCDHP. The committee collaborated to 1) create an emergency shelter audiovisual presentation that is repeatedly played on a portable DVD player at hurricane shelters and includes sign language, written words, and pictograms (29); 2) create an assistive technology definition sheet to assist emergency shelter managers in understanding how equipment can help someone maintain their independence; 3) create functional needs kits for persons with disabilities in public shelters, which included a small magnifier as a vision aid, a picture communication sheet as an augmentative communication aid, and washcloths and rubber bands to enlarge handles of items used to perform activities of daily living, such as brushing teeth and hair, writing, and eating; and 4) gather accessibility data on emergency shelters. To assess the emergency preparedness of persons with disabilities, in 2013 SCDHP added two questions to the South Carolina Behavior Risk Factor Surveillance System survey, a state-level survey that is part of the national surveillance system for monitoring the prevalence of behavioral risk factors among the population (30). The questions were focused on whether a person had an emergency kit and a disaster evacuation plan.

## Conclusions

Persons with disabilities can benefit from preventive and acute-care services in ways similar to persons without disabilities, yet they experience significant barriers to this care and health disparities when compared with persons who do not have disabilities. Prevention of disabilities has been the focus of public health, and prevention remains its primary focus, but as disability is acknowledged as part of the normal human experience, a secondary focus of public health has become the promotion of the health of persons with disabilities by identifying and closing reducible gaps between the health of persons with and without disabilities. A multifaceted approach is required to eliminate health disparities and reduce the socioeconomic disadvantages and structural barriers to the health system faced by persons with disabilities. Experiences at the local and state levels suggest that the key ingredients for success are building strong and long-lasting collaborations with diverse stakeholders and partners, identifying common goals, and integrating persons with disabilities into all facets of public health activities, including planning, surveillance, programming, education, and evaluation. Sustained support, including a mandate for programs and their surveillance systems to identify persons with disabilities, is crucial.
